# Effects of a 6-week, whole-body vibration strength-training on depression symptoms, endocrinological and neurobiological parameters in adolescent inpatients experiencing a major depressive episode (the “Balancing Vibrations Study”): study protocol for a randomized placebo-controlled trial

**DOI:** 10.1186/s13063-018-2747-8

**Published:** 2018-07-03

**Authors:** Max Oberste, Nicola Großheinrich, Heidrun-Lioba Wunram, Johannes Levin Graf, Alischa Ziemendorff, Axel Meinhardt, Oliver Fricke, Esther Mahabir, Stephan Bender

**Affiliations:** 1 Department of Child and Adolescent Psychiatry and Psychotherapy, Medical Faculty, University of Cologne, Robert-Koch-Straße 10, 50931 Cologne, Germany; 2 Department of Social Sciences, Catholic University of Applied Science of North Rhine – Westphalia, Wörthstraße 10, 50688 Cologne, Germany; 3 Chair of Child and Adolescent Psychiatry, Witten/Herdecke University and Department of Child and Adolescent Psychiatry, Psychotherapy and Child Neurology, Gemeinschaftskrankenhaus Herdecke, Gerhard-Kienle-Weg 4, 58313 Herdecke, Germany; 4 Comparative Medicine, Center for Molecular Medicine Cologne, University of Cologne, Robert-Koch-Str. 21, Cologne, Germany

**Keywords:** Exercise, Whole-body vibration, Major depression, Adolescence, Cortisol, Neurotrophins, Inflammatory markers

## Abstract

**Background:**

Moderate to vigorous endurance and strength-training exercise was suggested as a treatment option for major depression. However, there is little evidence to support this suggestion in adolescent patients. The present study investigates the effects of a whole-body vibration strength-training intervention on symptoms in medication-naïve adolescent inpatients experiencing a major depressive episode. Potential underlying endocrinological and neurobiological mechanisms are explored.

**Methods/design:**

A double-blinded randomized controlled trial is conducted at the University Hospital of Cologne in Germany, comparing a 6-week, whole-body vibration strength-training with a 6-week placebo-intervention, as add-on therapy to inpatient treatment as usual. Forty-one subjects (13–18 years of age) will be included in each of the two groups. The study is powered to detect (*α* = .05, *β* = .2) a medium effect size difference between the two groups (*d* = .5) in terms of patients’ change in the Children’s Depression Rating Scale raw-score, from baseline until the end of the intervention. As secondary endpoints, the effects of exercise treatment on patients’ cortisol awakening response as well as on brain-derived neurotrophic factor, insulin-like growth factor 1 and inflammatory markers (tumor necrosis factor-alpha, interleukin-6 and C-reactive protein) serum levels will be assessed.

**Discussion:**

This study will provide evidence on the effectiveness of whole-body vibration strength-training as an add-on therapy in adolescent inpatients experiencing a major depressive episode. After completion of data collection, the present study will be the largest randomized controlled trial so far to investigate the effectiveness of an exercise intervention in inpatient adolescents suffering from a major depressive episode. Moreover, the present study may help to determine the underlying mechanisms of potential anti-depressant effects of exercise in depressed adolescent inpatients.

**Trial registration:**

DRKS.de, German Clinical Trials Register (DRKS), Identifier: DRKS00011772. Registered on 20 March 2017.

**Electronic supplementary material:**

The online version of this article (10.1186/s13063-018-2747-8) contains supplementary material, which is available to authorized users.

## Background

Depression is one of the most common psychiatric diagnoses among adolescents [1, 2]. Prevalence rates of between 3 and 6% for meeting full criteria of major depressive disorder are reported [3–5]. Adolescent depression is particularly malignant. It increases the likelihood of recurrence and chronicity in adulthood [6, 7]. It is frequently accompanied by a wide range of psychiatric and somatic comorbidities [8–11] and typically leads to substantial impairment in social [12] and cognitive functioning [13], as well as in scholastic and vocational performances [13, 14]. The increased risk for suicide makes depression the third leading cause of death among adolescents worldwide [15].

In most countries, clinical guidelines recognize cognitive behavioral therapy and pharmacotherapy with the selective serotonin re-uptake inhibitor fluoxetine, or the combination of both, as evidence-based treatment options for adolescent depression [16–20]. However, cognitive behavioral therapy can be inaccessible and expensive [21, 22], whereas a clear advantage of pharmacotherapy for children and adolescents was recently questioned [23]. Pharmacotherapy entails possible side-effects [24]. Fluoxetine and venlafaxine were shown to be associated with an elevated risk of suicidal ideation and behavior (suicidality) in adolescents [25, 26]. Accordingly, alternative treatment approaches for adolescent depression are required. Recently, there has been a growing interest in physical activity as an alternative treatment option for depression. In adults suffering from a major depressive episode, a recent meta-analysis of 35 randomized controlled trials showed a moderate clinical effect of exercise treatments on depression symptoms [27]. Trials investigating the benefits of exercise interventions in adolescents experiencing a major depressive episode are rare [28], but first evidence is promising [29–31].

In a recent pilot study, our group showed comparable anti-depressant effects of a *whole-body vibration strength-training* (WBV-training) to an ergometer training intervention on a stationary cycle in adolescent stationary patients, when compared to *treatment as usual* (TAU). However, WBV-training was better accepted and patients reported higher motivation to maintain physical activity after discharge from the clinic compared to ergometer training [31]. Based on these findings, the present study continues to investigate WBV-training as a potential add-on therapy for adolescents suffering from a major depressive episode. The main objective of the present study is to replicate the anti-depressant effects of the 6-week WBV-training on depression symptoms in adolescent inpatients while also improving methodological shortcomings of our previous pilot study. Unlike in our pilot study, WBV-training is not tested against TAU, but against a *placebo control group intervention* (PCG-intervention) (supervised myofascial release training + TAU) providing a comparable amount of psychosocial stimulation. Moreover, in the present study, allocation to treatment groups is completely randomized. The severity of depression symptoms is not exclusively assessed by self-report, but also by blinded clinician rating. The statistical test power will be increased.

Several endocrinological and neurobiological adaptations to physical activity have been proposed to explain the anti-depressant effect of exercise interventions [32]. Emphasis is put on the positive effect of exercise on the hypothalamic-pituitary-adrenal axis (HPA axis) [33, 34], on neurotrophin [34, 35] and growth factor expression [36], as well as on inflammatory markers [34, 37, 38]. Changes in these parameters are supposed to promote beneficial structural and functional changes of the central nervous system via different pathways [39, 40], resulting in improvement of depression symptoms [34, 41]. However, existing research has been conducted almost exclusively in adults. Therefore, the secondary objective of the present study is to investigate endocrinological and neurobiological adaptations to the 6-week WBV-training. The effect of the WBV-training on HPA axis activity is assessed by measuring patients’ salivary cortisol awakening response [42]. WBV-training-induced changes in brain-derived neurotrophic factor (BDNF), insulin-like growth factor 1 and inflammatory markers (tumor necrosis factor-alpha, interleukin-6 and C-reactive protein (CRP)) serum levels are explored.

## Methods/design

**Fig. 1:**
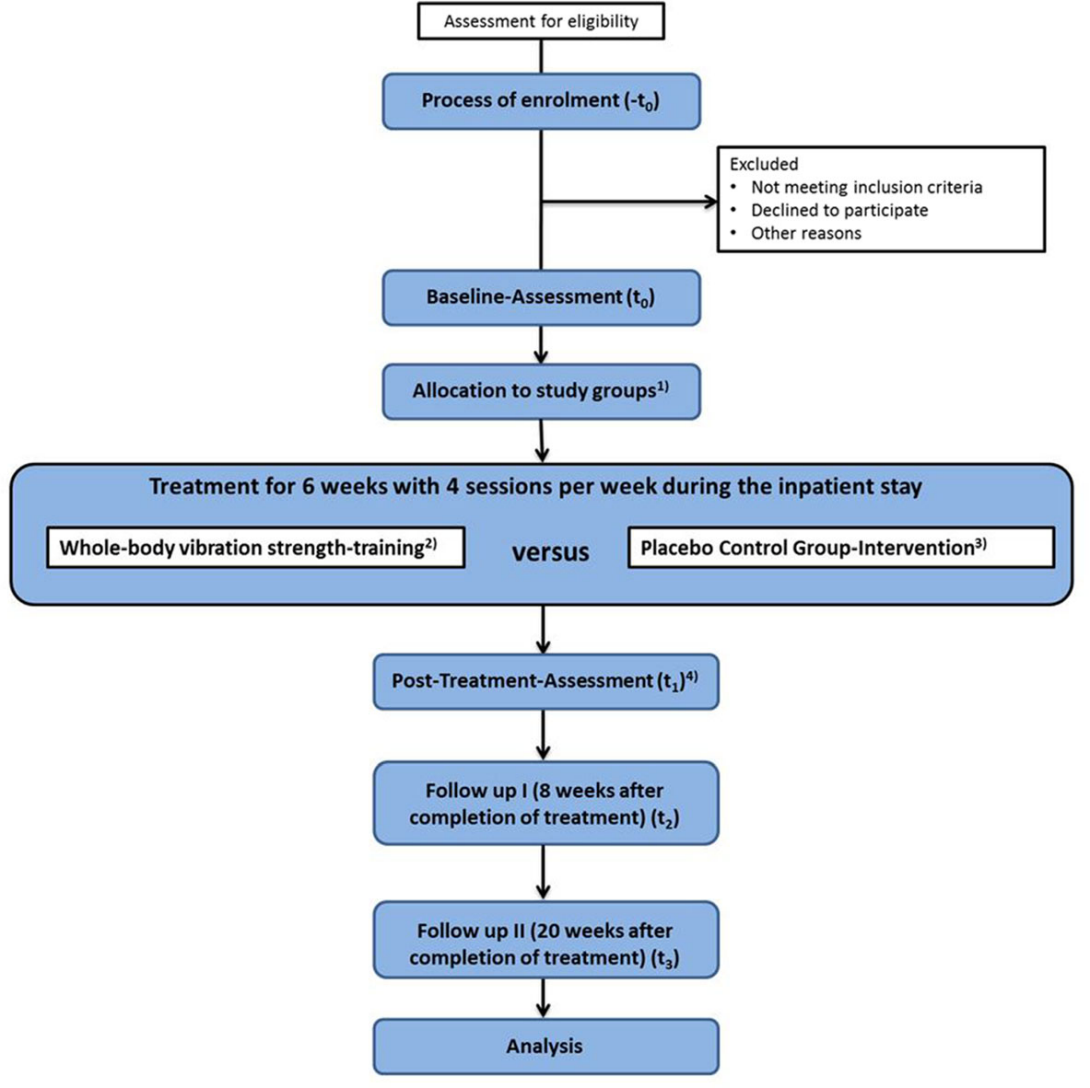
Flowchart of the trial. (1) A stratified block randomization with permuted block length is conducted to allocate patients into treatment groups. The stratification factor is patients’ gender (male vs. female); (2) Participants allocated to the whole-body vibration strength-training perform static and dynamic exercises on a Whole Body Vibration Plate; (3) Participants allocated to the Placebo Control Group receive a supervised myofascial release training program hardly inducing any muscular effort and cardiovascular stimulation; (4) Post-Treatment Assessment (t1) is conducted after completion of the 6-week exercise-treatment phase

The present study is designed as a longitudinal interventional study with two randomized treatment groups whose members will engage for 6 weeks in physical activity as add-on therapy to TAU (parallel-group, double-blinded, randomized controlled trial). The pre-specified objectives and hypotheses are listed in Table 1. The study flowchart is shown in Fig. 1. The study protocol was approved by the Ethics Committee of the University Hospital of Cologne (Germany) and is registered at the World Health Organization trial register (Identifier: DRKS00011772, see also Additional file 1). Any modifications to the protocol which may impact on the conduct of the study, potential benefit of the patient or may affect patient safety, including changes of study objectives, study design, patient population, sample sizes, study procedures, or significant administrative aspects will require a formal amendment by the ethics commission. The study conforms to the Declaration of Helsinki.

**Table 1:** Objectives and hypotheses of the study

Objectives	Hypotheses
Main objective • To evaluate the effect of a 6-week whole-body vibration-training vs. placebo on clinician-rated severity of depression symptoms in adolescent inpatients experiencing a major depressive episode	Main hypothesis • In adolescent inpatients experiencing a major depressive episode, the change in the Children’s Depression Rating Scale-Revised raw-scores from baseline to completion of the 6-week intervention differs significantly between the whole-body vibration strength-training group and the control group (hypothesis A)
Secondary objectives • To evaluate the effect of a 6-week whole-body vibration-training vs. placebo on hypothalamic-pituitary-adrenal axis dysregulation in adolescent inpatients experiencing a major depressive episode	Secondary hypotheses • In adolescent inpatients experiencing a major depressive episode, the change in cortisol awakening response from baseline to completion of the 6-week intervention differs significantly between the whole-body vibration-training group and the control group (hypothesis B)
• To evaluate the effect of a 6-week whole-body vibration-training vs. placebo on neurotrophin and growth factor expression in adolescent inpatients experiencing a major depressive episode	• In adolescent inpatients experiencing a major depressive episode, the change of brain-derived neurotrophic factor and insulin-like growth factor 1 serum levels from baseline to completion of the 6-week intervention/to 8 weeks’ follow-up differs significantly between the whole-body vibration-training group and the control group (hypothesis C)
• To evaluate the effect of a 6-week whole-body vibration-training vs. placebo on inflammatory-marker expression in adolescent inpatients experiencing a major depressive episode	• In adolescent inpatients experiencing a major depressive episode, the change in tumor necrosis factor-alpha, interleukin-6, C-reactive protein serum levels from baseline to completion of the 6-week intervention/to 8 weeks’ follow-up differs significantly between the whole-body vibration-training group and the control group (hypothesis D)
• To evaluate the effect of a 6-week whole-body vibration-training vs. placebo on self-perceived severity of depression symptoms in adolescent inpatients experiencing a major depressive episode	• In adolescent inpatients experiencing a major depressive episode, the change of Beck’s Depression Inventory-second edition raw-scores from baseline to completion of the 6-week intervention/to 8 weeks’ follow-up/to 20 weeks’ follow up differs significantly between the whole-body vibration-training group and the control group (hypothesis E)
• To evaluate the sustainability of the effect of the 6-week whole-body vibration-training vs. placebo on clinician rated severity of depression symptoms in adolescent inpatients experiencing a major depressive episode	• In adolescent inpatients experiencing a major depressive episode, the change of Children’s Depression Rating Scale-Revised raw-scores from baseline to 8 weeks’ follow-up/to 20 weeks’ follow-up differs significantly between the whole-body vibration-training and the control group (hypothesis F)

### Study population and recruitment

Participants are recruited from the adolescent psychiatric wards of the Department of Child and Adolescent Psychiatry and Psychotherapy at the University Hospital of Cologne, Germany. On admission to the inpatient treatment, adolescents and their parents (or other caregivers) are contacted by the study coordinator and informed about the program. If interest in participation is expressed, patients will be screened for eligibility. Inclusion and exclusion criteria of the study are listed in Table 2. Patients are included in the study only after they and their parents (or other caregivers) give written informed consent. The participants are included chronologically.

**Table 2:** Inclusion and exclusion criteria

Inclusion criteria	Exclusion criteria
• 13–18 years of age • Undergoing inpatient psychiatric treatment • Within the first 3 weeks of inpatient treatment • Meeting DSM-V and ICD-10 criteria of non-psychotic major depressive disorder assessed by clinician rating with the structured clinical interview ‘Schedule for Affective Disorders and Schizophrenia adapted for school-aged children (present and life-time)’ • Baseline raw-score of at least 40 points in the Children’s Depression Rating Scale-Revised	• Comorbid schizophrenia, personality disorder, autism spectrum disorder, schizoaffective disorder, acute or past psychotic events • Acute suicidality • Current substance abuse • Permanent medication with inherent psychotropic effects including fluoxetine • Non-substituted hypothyroidism • Addison’s disease • Body Mass Index less than 16 kg/m^2^ • Any contraindication that prevents participation in the exercise intervention • Insufficient knowledge of the German language • Intelligence quotient less than 70

*DSM-V Diagnostic and Statistical Manual of Mental Disorders, Fifth Edition*, *ICD-10 10th revision of the International Statistical Classification of Diseases and Related Health Problems*

### Randomization and blinding

Eligible patients are randomized to one of two treatments (see “Groups and treatments” section below). A stratified block randomization with permuted block length is conducted. The stratification factor is patients’ gender (male vs. female). Randomization of treatment groups is implemented based on sealed, opaque envelopes by the Institute of Medical Statistics and Computational Biology of the University of Cologne.

The present study is a double-blinded trial. Prior to participation, patients will be informed that two physical activity interventions (whole-body vibration strength-training/myofascial release training) will be compared regarding their effect on severity of depression symptoms in adolescent inpatients. Accordingly, patients do not know the specific hypotheses of the trial. They will not know if they are allocated to the experimental or to the control group and, therefore, can be considered blinded. Moreover, experimental testing procedures will be carried out by specially trained experimental staff that will also be blinded regarding the allocation of participants to the specific experimental group.

### Groups and treatments

WBV-training and PCG-intervention are conducted at the Department of Child and Adolescent Psychiatry and Psychotherapy at the University Hospital of Cologne. The participants of both intervention groups exercise four times per week for 6 weeks during their inpatient stay. Each session lasts approximately 30 *min*. The intervention sessions are carried out individually or in small groups with up to four participants. Training sessions are always instructed by specially trained study staff. In the following, WBV-training and PCG intervention will be described in detail:

- **Whole-body vibration strength-training**

Participants perform static and dynamic exercises on the Galileo® Whole Body Vibration Plate Med M (Novotec Medical GmbH, Pforzheim, Germany). The training sessions consist of six standardized exercises: squats, lunges, upper-body rotation against resistance, butterfly reverse against resistance, standing in partial squatting position, rocking the feet between the ball and heel. Resistance is applied using a latex resistance band (TheraBand, Akron, OH, USA). Each exercise is performed for 2 min. at 24 Hz and standing with feet parallel on the vibration plate, feet hip width apart. Between each exercise, participants stay active conducting low-intense shoulder and hip mobilization exercises for 2 min. After completion of four training sessions, vibration frequency is increased to 26 Hz. After completion of 12 trainings, the time of each exercise is increased to 3 min with 3-min. active rest intervals between the exercises.

- **Placebo control group intervention**

Patients allocated to the PCG-training receive a supervised myofascial release training program. The training sessions consist of seven standardized exercises using a foam roll (Blackroll, Bottighofen, Switzerland): self-massage of the soles of the feet (one leg stand), of the calves (lying in dorsal position), of the hamstrings (lying in dorsal position), of the thighs (manually while lying in dorsal position), of the neck (lying in dorsal position), of the lower and upper back (leaning backwards against a wall in standing position), as well as self-massage of the upper arm and shoulder (leaning sideways against a wall in standing position). Each exercise is repeated 10 times on each side of the body. Between each exercise, participants do low-intensity shoulder and hip mobilization exercises for 2 min. The PCG-intervention was designed to provide an amount of psychosocial stimulation comparable to the WBV-training. All myofascial release exercises are instructed and patients are corrected, if necessary. However, unlike the WBV-training, myofascial release training sessions applied to the PCG hardly induce any muscular effort and cardiovascular stimulation.

Contraindications for participation in a training session in the WBV-training and in the PCG-intervention are acute suicidality, injuries, infections, or fever. To increase adherence to the training, participants are picked up from the ward by study staff. Treatment participation and comments on each session will be recorded.

The WBV-training and PCG-intervention are conducted simultaneously with the patients’ TAU at the Department of Child and Adolescent Psychiatry and Psychotherapy at the University Hospital of Cologne. TAU is carried out by a multidisciplinary team, consisting of psychiatrists, psychotherapists, music therapists, art therapists, pedagogs and social workers. Patients receive psychotherapy in the form of individual sessions one to two times per week. Families are closely involved in the patients’ therapy process. Every 4 weeks, systemic family therapy sessions are conducted. In addition, patients participate in individual and group music-therapy and art-therapy sessions. Counseling by the social services is conducted once or twice during inpatient stay.

### Data collection and timeline

**Fig. 2:**
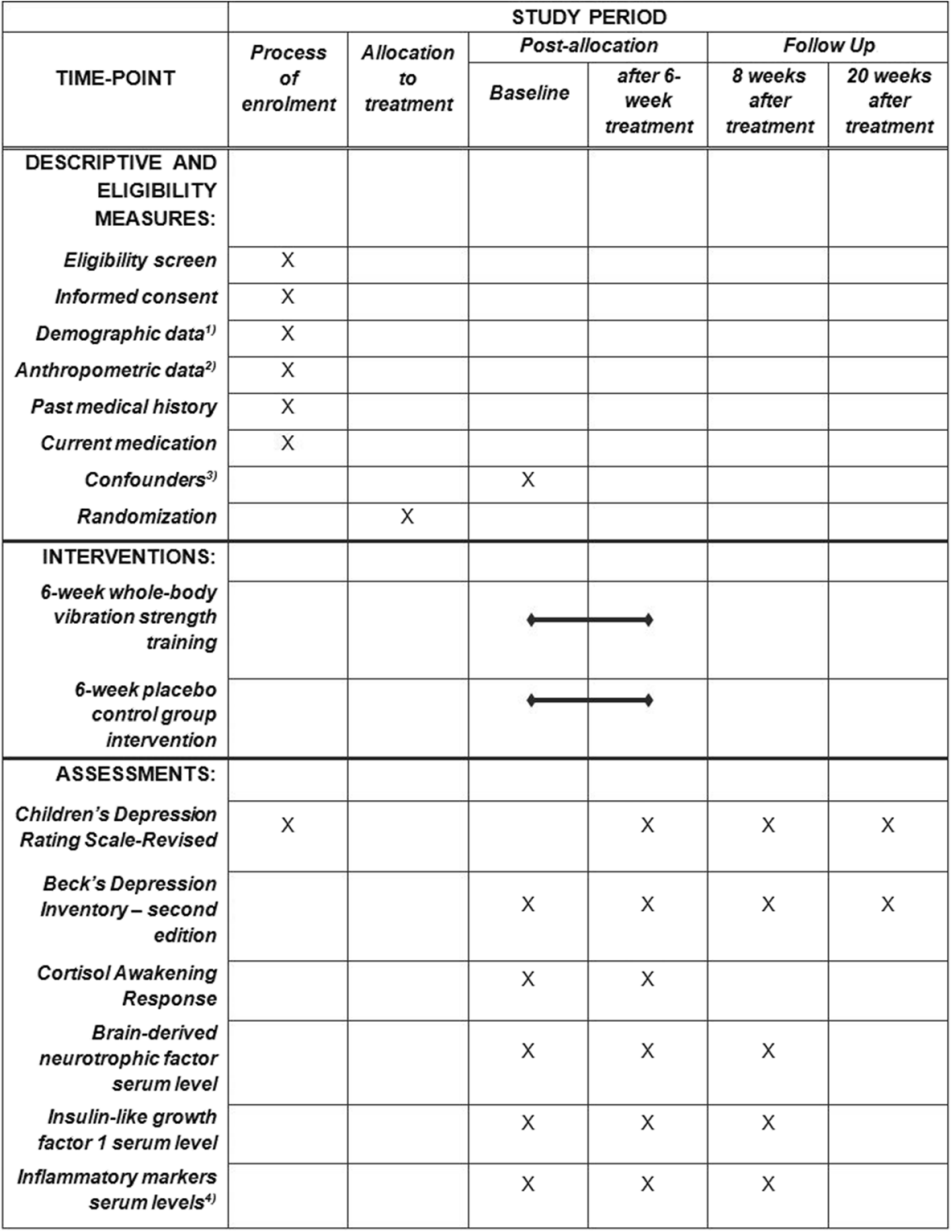
The Standard Protocol Items: Recommendations for Interventional Trials (SPIRIT) schedule of enrollment, interventions and assessments. (1) Demographic data will be assessed: age, sex and education; (2) Anthropometric data will be assessed: height, weight and BMI; (3)Potential confounding factors will be assessed: current psychiatric comorbidities, as well as patients’ history of mental disorders (Schedule for Affective Disorders and Schizophrenia-Present and Lifetime Version adapted for school-aged children, Diagnostic System for Mental Disorders in Childhood and Adolescence), emotional and/or behavioral problems (Child Behavior Checklist/4–18, Youth Self-Report/11–18) and socioeconomic status (modified questionnaire from Trinkl and colleagues [51]); (4) Inflammatory markers will be assessed: tumor necrosis factor-alpha, interleukin-6 and C-reactive protein are assessed

Data are collected *during the process of enrollment* (− t_0_), *at baseline* (t_0_), *after completion of the 6-week intervention* (t_1_), *8 weeks after completion of the 6-week intervention* (follow-up I, t_2_), as well as *20 weeks after completion of the 6-week intervention* (follow-up II, t_3_). At –t_0_, patients’ demographic (age, sex, education, socioeconomic status) and anthropometric data (weight, height, Body Mass Index (BMI)), as well as past medical history and current medication, are collected. To determine patients’ eligibility for study participation before allocation to treatment groups, the primary outcome measure of the planned study (the *Children’s Depression Rating Scale-Revised* (CDRS-R)) is also assessed at − t_0_. A raw-score of 40 or more points in the CDRS-R must be present to take part in the study [43] (compare Table 2). Baseline measurement takes place 1–3 days after enrollment. At t_0_, all secondary outcome measures of the study (see “Secondary endpoints” section below), as well as potential confounding factors (see “Potential confounding factors” section below), are protocolled. At t_1_, the primary outcome measure and all secondary outcome measures are assessed. The t_1_ measurement takes place 1–3 days after completion of the 6-week exercise-treatment phase. At t_2_, the primary outcome measure and all secondary outcome measures of the study, except cortisol awakening response, are assessed. Finally, at t_3_, only the CDRS-R and the *Beck’s Depression Inventory -second edition* (BDI-II) are applied. Time accuracy of both follow-up intervals lies within a range of ± 3 days. The Standard Protocol Items: Recommendations for Interventional Trials (SPIRIT) study schedule of enrollment, interventions and assessments is provided in Fig. 2. The SPIRIT Checklist [44] is provided as Additional file 2 to this publication.

### Primary endpoint

The primary endpoint of the present study is patients’ change of severity of depression symptoms from baseline until the end of the 6-week intervention. Patients’ severity of depression symptoms is measured by the CDRS-R. In the context of clinical trials, the CDRS-R is one of the most frequently used clinician ratings for the assessment of depressive symptomatology in adolescents [45]. The CDRS-R is a semi-structured interview consisting of 17 items that are rated between 1 (“no difficulties”) and 5 or 7 points (“clinically significant difficulties”). Usually, a raw-score of 40 points is used as a cut-off for depressive symptomatology and higher raw-scores are interpreted as more severe depression symptoms [43]. For CDRS-R application in adolescents, excellent internal consistency and good convergent validity have been reported [45].

### Secondary endpoints

The following secondary endpoints are assessed in the present study:

- **Hypothalamic-pituitary-adrenal axis activity**

HPA axis activity is assessed by measuring patients’ cortisol awakening response. Saliva samples are collected after forced awakening at 7.00 and at 7.30 a.m. on two consecutive weekdays at t_0_ and at t_1_. Patients are asked to remain in bed without going back to sleep and to refrain from drinking and eating during the 30-min sampling period. In case of non-adherence (i.e., awakening before forced waking, rising before the second sample is collected, eating, or drinking), the procedure is repeated on the following day. Saliva is collected using Salivette collection devices (Sarstedt, Nümbrecht, Germany). Saliva samples are frozen and stored at − 20 °C until analysis. After thawing, salivettes are centrifuged at 3000 rpm for 5 min. Salivary cortisol concentrations are measured using commercially available chemiluminescence immunoassay with high sensitivity (IBL International, Hamburg, Germany). The intra- and interassay coefficients for cortisol are required to be below 8%. To measure HPA axis activity, mean salivary cortisol concentration and the increase between baseline and the 30-min samples are determined.

- **Serum brain-derived neurotrophic factor, insulin-like growth factor 1 and inflammatory-marker levels**

Drawing of 8.5 ml venous blood from sedentary patients is performed at t_0_, t_1_ and t_2_. Samples for BDNF, cytokine (insulin-like growth factor-1, tumor necrosis factor-alpha, interleukin-6) and CRP analysis will be taken from a peripheral vein of the arm and collected in serum gel tubes (S-Monovette® Serum, Sarstedt, Nümbrecht, Germany). After complete coagulation at 4 °C, samples will be centrifuged for 10 min. at 1000 g and 4 °C. The serum will be prepared and aliquoted within 24 h after blood drawing. Duplicate samples will be stored at − 80 °C until analyzed when samples of all included participants are collected. Serum levels of BDNF and cytokines are assessed at t_0_, t_1_ and t_2_. BDNF and cytokines will be measured in duplicates in a multiplex analyzer (Bio-Plex 200®, Bio-Rad Laboratories) according to the manufacturer’s instructions. Sera will be thawed, centrifuged for 10 min. at 10,000 g and 4 °C and diluted in sample diluent (1:4). By using the median of the fluorescence intensity and the standard curve, the absolute concentration of BDNF and the cytokines (pg/ml) will be calculated (Bio-Plex Manager 6.1, Bio-Rad Laboratories). For determination of CRP, the blood will be drawn as described above in lithium-heparin tubes (S-Monovette®, lithium-heparin, Sarstedt, Nümbrecht, Germany). It will be centrifuged for 10 min. at 4000 g and 21 °C. Plasma will be aliquoted within 3 h after blood drawing and used fresh. The CRP level will be determined via latex agglutination assay according to the manufacturer’s instructions (C-Reactive Protein Gen.3, cobas®, Roche Diagnostics, Basel, Switzerland). Briefly, plasma will be diluted 1:100 and added on a slide, which is pre-coated with antibodies to monoclonal anti-human CRP and latex reagent. After 2 min incubation, clear agglutination will be observed on the slide and it will be examined turbidimetrically using the analytic system cobas® C702 (Roche Diagnostics). CRP values below 3 mg/l are considered clinically irrelevant and will be adjusted to 0 mg/l.

- **Self-rated severity of adolescent patients’ depression symptoms**

For the self-rating procedure, the German version of the BDI-II [46] is applied. The BDI-II was developed as an indicator of the presence and severity of depression in psychiatric patients from 13 years of age onwards. Twenty-one symptoms are rated by the patient from 0 to 3, with higher scores indicating more severe occurrence of the particular symptom. Accordingly, the sum score ranges from 0 to 63, with higher scores indicating more severe depressive symptomatology. The BDI-II is sensitive for changes in severity of depressive symptomatology. Excellent indices of reliability, as well as good discriminant and convergent validity, have been reported in adolescent psychiatric inpatients [47].

- **Sustainability of effects of whole-body vibration strength-training on severity of depression symptoms**

CDRS-R is additionally applied 8 and 20 weeks after completion of the treatment to assess sustainability of potential effects of WBV-training compared to placebo.

The “Balancing Vibrations Study” also comprises magnetic resonance imaging, as well as combined transcranial magnetic stimulation and electroencephalographic recordings. For clarity, the more complex protocols and hypotheses, regarding these neuroimaging/neurophysiological parameters, will be illustrated separately and reported elsewhere.

### Potential confounding factors

Several additional factors, potentially impacting the primary outcome of the study (severity of depression symptoms), are assessed at t_0_. To confirm depression diagnosis and to capture current psychiatric comorbidities, as well as patients’ history of mental disorders, the “Schedule for Affective Disorders and Schizophrenia – Present and Lifetime Version adapted for school-aged children” [48] and the “Diagnostic System for Mental Disorders in Childhood and Adolescence” [49] are applied. For assessment of emotional and/or behavioral problems, the “Child Behavior Checklist/4–18,” as well as the “Youth Self-Report/11–18” of the Achenbach System of Empirically Based Assessment, are used [50]. The patients’ socioeconomic status is assessed with a modified questionnaire developed by Trinkl and colleagues [51].

### Adverse events and compliance assessment

Adverse events and serious adverse events, which are related to the interventions, are assessed by the clinicians. During our pilot study, neither adverse events nor severe adverse events occurred [31]. Compliance to WBV-training and PCG-intervention is protocolled by scientific staff. It was pre-defined that patients need to participate in 20 sessions for complete compliance. This was shown feasible in our pilot study [31].

The Department of Child and Adolescent Psychiatry and Psychotherapy at the University Hospital of Cologne has insurance to cover for non-negligent harm associated with the protocol. This will include cover for additional health care, compensation, or damages. Incidences judged to arise from negligence (including those due to major protocol violations) will not be covered by study insurance policies.

### Data management and confidentiality

All data will be entered electronically by two independent student assistants. Any deviations will be clarified and corrected. Original study forms will be kept on file. All study-related information is stored in a secure and accessible place and manner. All participant information will be stored and locked in file cabinets in areas with limited access. Participant files will be kept in storage for a period of 3 years after completion of the study. Blood samples will be disposed after analyses.

All laboratory samples, reports, data collection, process and administrative forms will be identified by a coded identification number, to maintain participant confidentiality. All records that contain names or other personal identifiers, such as locator forms and informed consent forms, will be stored separately from study records, which are also identified by code numbers. All local databases will be secured with password-protected access systems. Forms, lists, logbooks, appointment books and any other listings that link participant identification numbers to other identifying information will be stored in a separate, locked file in an area with limited access.

### Sample size calculation

Sample size calculation was done for the effect of experimental treatment (WBV-training vs. PCG intervention) on the primary endpoint (severity of depression symptoms) measured by CDRS-R raw-scores in an *analysis of covariance* (ANCOVA) model using G*Power 3.1.9.2 software [52]. It was hypothesized that changes in the CDRS-R raw-scores from baseline to completion of the 6-week intervention differ significantly between the WBV-training group and the PCG-training group. As clinically relevant effect size, a medium effect of *d* = .5 was defined. Alpha was set at 5%. Test power (1 − *β*) was set at 80%. Participants’ CDRS-R scores at baseline will be included in the model as covariate. Required sample size in such an ANCOVA model can be calculated as (1 − *ρ*^2^) × *n*, with *ρ* representing the correlation between participants’ baseline and post-treatment outcome scores, and *n* representing the sample size that would have been required if a *t* test of post-treatment outcome scores was applied [53]. Based on the results of our pilot study, we estimated the correlation between patients’ severity of depression symptoms at baseline and at post treatment with *ρ* = .6. Under the presuppositions made, sample size calculation showed that 41 patients in each group (*N* = 82) would be required. We accounted for a 15% dropout rate, leading to a total sample size of *N* = 94 patients.

### Statistical analysis

An intention-to-treat analysis will be performed. We will also assess the effect of the complete treatment in a per-protocol analysis. To determine the effects of between-subjects factor “treatment,” within-subjects factor “measurement time point” and their interaction on primary and secondary outcomes, separate 2 × 3 (hypothesis A, E, F)/2 × 2 (hypothesis C, D) mixed ANCOVAs will be conducted. For secondary outcome “HPA axis activity” (hypothesis B) separate 1-factor ANCOVA will be conducted. As covariate, patients’ baseline scores of each specific parameter will be used. A significant main effect of within-subjects factor measurement time point will be further investigated through post hoc pairwise comparisons. Simple effects analyses will be conducted to determine potential group differences at each time point. Due to the exploratory character of the secondary hypotheses, alpha error adjustment will not be applied.

ANCOVA assumptions will be explored. In case of violations of normality assumption, appropriate non-parametric procedures will be used. In case of violations of ANCOVA assumptions of homogeneous variances for between-subjects factor levels and sphericity for variances of differences between categories of within-subjects factor, the F-test will be adjusted. Eta-squared values will be reported as effect size estimates for explained variance, and Cohen’s *d* values will be reported for post hoc pairwise comparisons.

Statistical analysis will be performed by a statistician, blinded to treatment groups. Statistical analyses will be conducted using SPSS 25® (IBM®, Armonk, NY, USA). A result will be considered significant at a *p* value equal to or less than .05.

## Discussion

Research on the anti-depressant effects of physical activity in clinically depressed adolescent patients is rare. Apart from our previously published pilot study [31], to the best of our knowledge, only two small trials examined the effects of exercise interventions in clinically depressed adolescent patients [29, 30]. The latter two studies exclusively included ambulatory patients. Our pilot study was the first to investigate the anti-depressant effects of an exercise intervention on depression symptoms in adolescents in an inpatient setting. The first results are promising. Anti-depressant effects, comparable to those reported in clinically-depressed adults [27], were shown. However, methodological shortcomings; e.g., small sample sizes, incomplete randomization, lack of placebo treatment in the control group, and only self-report of depressive symptoms, reduce the validity of the existing results. To incorporate physical activity into treatment of adolescent depression, more clinical randomized controlled trials at a high methodological level are needed. After completion of data collection, the present study will be the largest randomized controlled trial so far to investigate add-on exercise therapy in inpatient adolescents experiencing a major depressive episode.

Beyond the investigation of anti-depressant effects of WBV-training on clinical depression symptoms in adolescent inpatients, the present study explores potential underlying physiological mechanisms. To reliably attribute changes in clinical depression symptoms, after an exercise intervention to physiological adaptations to the physical activity, exercise treatment and control group treatment must fulfill the *ceteris paribus* clause. This means that the exercise group and the control group should be equal regarding all factors except for the critical ingredient (here: the physical exertion) [54, 55]. The present study compares the effects of WBV-training, not just against TAU, but against a placebo control group. WBV-training and PCG-training are both supervised and, therefore, exhibit a comparable amount of social attention to the patients. Accordingly, it will be possible to disentangle the effects of physiological factors from the potential effects of psychosocial factors related to the experimental treatment on patients’ depression symptoms. Thus, placebo and Hawthorne effects can be avoided.

The knowledge about the endocrinological and neurobiological mechanisms potentially underlying the anti-depressant effects of exercise is still in its infancy. Moreover, existing research has been conducted almost exclusively in adults. Our study will contribute to filling this knowledge gap. The present study investigates the effects of WBV-training on HPA axis activity, neurotrophin expression and inflammatory-marker expression. Hyperactivity of the HPA axis in major depression is one of the most consistent and robust biological findings in psychiatry. Hyperactivity of the HPA axis results in hypercortisolemia [56]. Continuously increased levels of cerebral cortisol lead to increased brain inflammation [57]. The anti-inflammatory effect of acute cortisol secretion changes with chronic exposure to a pro-inflammatory effect [58, 59]. Chronic glucocorticoid exposure and increased levels of CRP and pro-inflammatory cytokines, such as tumor necrosis factor-alpha and interleukin-6, within the central nervous system are associated with reduced levels of neurotrophins, like BDNF [60]. First evidence indicates that exercise positively influences cortisol levels in depressed adolescents [37], but further research is required. In adults exhibiting depression symptoms, neuroimmunomodulatory effects [61] and increases in neurotrophins due to exercise training have been reported [62]. It remains unclear if WBV-training in clinically depressed adolescents has similar molecular effects.

## Trial status

The recruitment of the patients started on 1 August 2017 and is still ongoing. Recruitment is expected to be completed by 31 July 2020. The data analysis and the writing of scientific manuscripts will be carried out after completion of recruitment.

## Additional files

Additional file 1: All Items from the World Health Trial Registration Data Set. (PDF 80 kb)

Additional file 2: Standard Protocol Items: Recommendations for Interventional Trials (SPIRIT) 2013 Checklist. (PDF 129 kb)

## Abbreviations

ANCOVA: Analysis of covariance
BDI-II: Beck’s Depression Inventory-second edition
BDNF: Brain-derived neurotrophic factor
CDRS-R: Children’s Depression Rating Scale-Revised
CRP: C-reactive protein
DRKS: German Clinical Trials Register
HPA axis: Hypothalamic-pituitary-adrenal axis
PCG-intervention: Placebo control group intervention
SPIRIT: Standard Protocol Items: Recommendations for Interventional Trials
t_0_: Baseline measurement
− t_0_: Time point of enrollment
t_1_: After completion of the 6-week intervention
t_2_: 8 weeks after completion of the 6-week intervention; follow-up I
t_3_: 20 weeks after completion of the 6-week intervention; follow-up II
TAU: Treatment as usual
WBV-training: Whole-body vibration training
