# Supplementary material for: Effects of a 6-week, whole-body vibration strength-training on depression symptoms, endocrinological and neurobiological parameters in adolescent inpatients experiencing a major depressive episode (the “Balancing Vibrations Study”): study protocol for a randomized placebo-controlled trial
Source: Trials. 2018 Jul 3;19:347. doi: 10.1186/s13063-018-2747-8 (PMC6029053; doi:10.1186/s13063-018-2747-8)
Supplement: Supplementary file 1 — All Items from the World Health Trial Registration Data Set. (PDF 80 kb) [file 13063_2018_2747_MOESM1_ESM.pdf]

Deutsches Register Klinischer Studien
German Clinical Trials Register

Trial Description

Title

Randomized controlled effects of a whole body vibration training on clinical symptoms and neurobiology in adolescent inpatients with major depressive episode

Trial Acronym

Balancing Vibrations

URL of the trial

http://neurologie-psychiatrie.uk-koeln.de/kinder-und-jugendpsychiatrie/klinik/stationaere-versorgung/jugendstation-1-2

Brief Summary in Lay Language

The positive effects of exercise in adult depression has already be found in previous studies. In our pilot study "Mood Vibes" we could demonstrate that high-intensity training with vibration devices ("whole body vibrations – WBV) has the same antidepressant effects as endurance sport in depressive adolescents. In the present study "Balancing Vibrations", we want to confirm these data on a bigger sample (n=82) and compare to a placebo sport condition. Moreover, the biological effects (neurophysiological, neuroneogenetical, anti-inflammatory, neurocognitive) of whole body vibration (WBV) exercise will be understood in a better way. WBV is mainly used for muscular/ strength training in rehabilitation clinics, but is also well established as a training device ("powerplate") in fitness centers.

We have changed the title of the study on 2nd of February 2018 to make clearer the entire spectrum of our investigations. The ethics committee was informed.

We decribed the intervention and the CG treatment more precisely on 2nd of February 2018. Participants are supposed to attend the training at least 4 times but they can also attend 5 times.

At the 2nd of February 2018 the primary endpoint was reduced to the expert rating and the self rating was made a secondary endpoint.

At the 2nd of February 2018 the measurements were slightly adjusted. The ethivs committee was informed.

At the 2nd of February 2018, the minimum age was changed to 13 years. Till that change no participants below 13 years of age had been included. Lifting the minimum age allows to capture self rated depression symptoms with BDI which is normed from age 13.

At the 2nd of February 2018, the targeted sample size was reduced to 82. The primary research question is about change in CDRS scores. This can be evaluated best with an ANCOVA model using baseline values as covariate. In such a model, a mean difference of d=.5 can be shown with a probability of 80 % (p=.05) in a sample of 82 (equally distributed in the two groups).

Brief Summary in Scientific Language

Antidepressant effects of high-frequent exercise (whole body vibration) as add-on therapy to "treatment as usual". Underlying mechanisms of action of exercise therapy in depression. Neurophysiological differences between short-term effects of a single exercise intervention and long-term therapeutic effects of a 6 weeks training program.

Organizational Data

- DRKS-ID: **DRKS00011772**
- Date of Registration in DRKS: **2017/03/20**
- Date of Registration in Partner Registry or other Primary Registry: **[---]***
- Investigator Sponsored/Initiated Trial (IST/IIT): **yes**
- Ethics Approval/Approval of the Ethics Committee: **Approved**
- (leading) Ethics Committee Nr.: **15-426 , Ethik-Kommission der Medizinischen Fakultät der Universität zu Köln**

Secondary IDs

- Universal Trial Number (UTN): **U1111-1193-4116**

Health condition or Problem studied

- ICD10: **F32 - Depressive episode**
- ICD10: **F33 - Recurrent depressive disorder**

Interventions/Observational Groups

- Arm 1: **Whole Body Vibration: 4-5 times week, 30 minutes each intervention, duration of Intervention 6 weeks.**
- Arm 2: **Placebo-condition (myofascial relaxation training): 4-5 times a week for 30 minutes. No muscular activation, intervention focus on personal attention and social factors of sports. Duration: 6 weeks (as WBV Group)**

Characteristics

- Study Type: **Interventional**
- Study Type Non-Interventional: **[---]***
- Allocation: **Randomized controlled trial**
- Blinding: **[---]***
- Who is blinded: **patient/subject, investigator/therapist, assessor, data analyst**

Study Type: **Interventional**

Study Type Non-Interventional: **[---]***

Allocation: **Randomized controlled trial**

Blinding: **[---]***

Who is blinded: **patient/subject, investigator/therapist, assessor, data analyst**

- Control: **Placebo**
- Purpose: **Treatment**
- Assignment: **Parallel**
- Phase: **N/A**
- Off-label use (Zulassungsüberschreitende Anwendung eines Arzneimittels): **N/A**

Primary Outcome

- t0, t1 (afters 6 weeks), t2 (after 14 weeks), t3 (after 26 weeks): CDRS-R (Children Depression Rating scale, blinded clinician Rating)

Secondary Outcome

- t0, t1, t2, t3: Beck' depression inventory (BDI)
t0, t1: Saliva Cortisol
- t0, t1, t2:: (IL-6, TNF Alpha), BDNF, IGF 1 (Blood),
- t0, t1, t2: Neurocognition (CANTAB®)
- t0, t1, t2: other questionnaires (CTQ (Childhood Trauma questionnaire), EZK (Eigenzustandsskala), WKV (wahrgenommene körpergebunden Verfassung), MSES (Magglinger Sport Enjoyment Scale), CBCL (Child Behavior Checklist), YSR (Youth Self Report), JTCI(Junior Temperament Character Inventory)
- t0, after first training session, t1, t2: neurophysiology (TMS-EEG)
- t0, t2: cMRI
- t0 BDNF Polymorphisms

Countries of recruitment

- DE **Germany**

Locations of Recruitment

- Medical Center **Kinder- und JUgendpsychiatrie der Uniklink Köln, Köln**

Recruitment

- Planned/Actual: **Actual**
- (Anticipated or Actual) Date of First Enrollment: **2017/05/22**
- Target Sample Size: **82**
- Monocenter/Multicenter trial: **Monocenter trial**
- National/International: **National**

Inclusion Criteria

- Gender: **Both, male and female**
- Minimum Age: **13 Years**
- Maximum Age: **18 Years**

Additional Inclusion Criteria

- participants have to meet ICD-10 criteria of depressive disorder (ICD-F32,ICD-F33)
- recidivating depressive disorder
- bipolar disorder with actual depressive Episode without psychotic symptoms
- age 13 to 18
- informed consent from participants and participants' parents
- no medication or on stable Long-term medication

Exclusion criteria

- schizophrenia, personality disorders, autism spectrum disorders, other psychotic disorders, schizo-affective disorder, acute suicidality
- unsufficient german language skills
- IQ < 70
- Epilepsy (of subject current or in medical history, or of relatives
- serious head injuries and head sugeries
- Current substance abuse
- Body Mass Index < 16 kg/sqm
- diseases causing restrictions to physical activity and the use of vibration devices, malignant diseases
- permanent long-term psychiatric medication or medication with inherent psychotropic effects (anticonvulsants, steroids, methylphenidate, amphetamine, antidepressants, neuroleptics, benzodiazepines, mood-stabilizer)
- Patients with Morbus Addison or non-substituted hypothyroidism
- Current pregnancy
- metal implants (e.g. pacemaker implantation)
coffee and cigarette consumption 24 hours until neurophysiological investigations start
- intake of stimulants medication the day of Investigation or 48 h Prior to neurophysiological and MRI Investigation in case of Retard medication

Addresses

- **Primary Sponsor**

  Uniklinik Köln, Klinik und Poliklinik für Psychiatrie, Psychosomatik und Psychotherapie des Kindes- und Jugendalters
  Robert-Koch-Str. 10
  50931 Köln
  Germany

  Telephone: **0221-478-5337**
  Fax: **[---]***
  E-mail: **kjp-depression at uk-koeln.de**
  URL: **http://neurologie-psychiatrie.uk-koeln.de/kinder-und-jugendpsychiatrie**

- **Contact for Scientific Queries**

  Uniklinik Köln, Klinik für Kinder- und Jugendpsychiatrie und -psychotherapie
  Ms. Dr. Heidrun-Lioba Wunram
  Robert-Koch-Straße 10
  50931 Köln
  Germany

  Telephone: **0221-478/ -98642/97316**
  Fax: **[---]***
  E-mail: **heidrun-lioba.wunram at uk-koeln.de,**
  URL: **[---]***

- **Contact for Public Queries**

  Uniklinik Köln, Klinik für Kinder- und Jugendpsychiatrie und -psychotherapie
  Mr. Dipl. Sportwiss., BA Psych. Max Oberste
  Robert-Koch-Straße 10
  50931 Köln
  Germany

  Telephone: **+49-221-478-5337**
  Fax: **+49-221-478-3428**
  E-mail: **max.oberste at uk-koeln.de**
  URL: **http://neurologie-psychiatrie.uk-koeln.de/kinder-und-jugendpsychiatrie/kontakt-und-anfahrt**

- **Collaborator, Other Address**

  Kinder- und Jugendpsychiatrie
  Mr. Prof. Dr. Stephan Bender
  Robert-Koch-Straße
  10 Köln
  Germany

  Telephone: **[---]***
  Fax: **[---]***

Collaborator, Other Address

Kinder- und Jugendpsychiatrie
Mr. Prof. Dr. Stephan Bender
Robert-Koch-Straße
10 Köln
Germany

Telephone: **[---]***
Fax: **[---]***
E-mail: **stephan.bender at uk-koeln.de**
URL: **[---]***

- **Collaborator, Other Address**

Katholische Hochschule NRW
Ms. Prof. Dr. Nicola Grossheinrich
Robert-Schumann-Straße 25
52066 Aachen
Germany

Telephone: **[---]***
Fax: **[---]***
E-mail: **n.grossheinrich at katho-nrw.de**
URL: **[---]***

- **Collaborator, Other Address**

Gemeinschaftskrankenhaus Herdecke, Klinik für Kinder- und Jugendpsychiatrie
Mr. Prof. Dr. Oliver Fricke
Gerhard-Kienle-Weg 4
58313 Herdecke
Germany

Telephone: **0 23 30 62-3406**
Fax: **0 23 30 62-3338**
E-mail: **o.fricke at gemeinschaftskrankenhaus.de**
URL:
http://www.gemeinschaftskrankenhaus.de/de/fachabteilungen/kinder.jugendp

- **Collaborator, Other Address**

Uniklinik Köln, Klinik und Poliklinik für Kinderheilkunde und Jugendmedizinund UniReha GmbH Zentrum für Prävention und Rehabilitation
Mr. Prof. Dr. Eckhard Schönau
Kerpener Str. 62
50937 Köln
Germany

Telephone: **+49 221 478-84755**

Collaborator, Other Address

Uniklinik Köln, Klinik und Poliklinik für Kinderheilkunde und Jugendmedizinund UniReha GmbH Zentrum für Prävention und Rehabilitation
Mr. Prof. Dr. Eckhard Schönau
Kerpener Str. 62
50937 Köln
Germany

Telephone: **+49 221 478-84755**
Fax: **+49 221 478-1424360**
E-mail: **eckhard.schoenau at uk-koeln.de**
URL: **https://kinderklinik.uk-koeln.de/erkrankungen-therapien/endokrinologie-stoffwechselerkrankungen/**

- **Collaborator, Other Address**

Deutsche Sporthochschule KölnInstitut für Kreislaufforschung und Sportmedizin
Mr. Prof. Dr. Wilhelm Bloch
Am Sportpark Müngersdorf 6
50933 Köln
Germany

Telephone: **+49-221-4982-5380**
Fax: **+49-221-4912906**
E-mail: **w.bloch at dshs-koeln.de**
URL: **https://www.dshs-koeln.de/visitenkarte/person/univ-prof-dr-wilhelm-bloch/**

- **Collaborator, Other Address**

Head Research Group Comparative MedicineHead CMMC Animal FacilityCenter for Molecular Medicine, University of Cologne (CMMC)
Ms. PD Dr. Esther Mahabir-Brenner
CMMC Research Building, Bldg. 66, Robert-Koch-Str. 21
50933 Köln
Germany

Telephone: **+49-221 478 89540**
Fax: **+49-221 478 3984**
E-mail: **Esther.Mahabir-Brenner at uni-koeln.de**
URL: **http://www.cmmc-uni-koeln.de/home/**

- **Collaborator, Other Address**

Lehrstuhl für Biologische Psychologie, Universität Dresden
Mr. Prof. Dr. Clemens Kirschbaum
Zellescher Weg 19
01069 Dresden
Germany

Collaborator, Other Address

Lehrstuhl für Biologische Psychologie, Universität Dresden
Mr. Prof. Dr. Clemens Kirschbaum
Zellescher Weg 19
01069 Dresden
Germany

Telephone: **[---]***
Fax: **[---]***
E-mail: **[---]***
URL: **[---]***

- **Collaborator, Other Address**

IMSIE- Institut für Medizinische Statistik, Informatik und Epidemiologie
Mr. Prof. Dr. Martin Hellmich
Universitätsklinikum Köln (AöR) - Gebäude 22
50937 Köln
Germany

Telephone: **+49 (0)221 478-6517**
Fax: **+49 (0)221 478-6520**
E-mail: **martin.hellmich at uni-koeln.de**
URL: **www.imsieweb.uni-koeln.de**

Sources of Monetary or Material Support

- **Private sponsorship (foundations, study societies, etc.)**

Marga und Walter Boll-Stiftung
Marie-Curie-Str. 8
50170 Kerpen
Germany

Telephone: **[---]***
Fax: **[---]***
E-mail: **[---]***
URL: **www.bollstiftung.de**

Status

- Recruitment Status: **Recruiting ongoing**
- Study Closing (LPLV): **[---]***

Trial Publications, Results and other documents

** This entry means the parameter is not applicable or has not been set.*
**** This entry means that data is not displayed due to insufficient data privacy clearing.*
